# The pathological mechanism of the COVID-19 convalescence and its treatment with traditional Chinese medicine

**DOI:** 10.3389/fphar.2022.1054312

**Published:** 2023-01-10

**Authors:** Linlin Jiang, Xuedong An, Yingying Duan, Fengmei Lian, De Jin, Yuehong Zhang, Cunqing Yang, Yuqing Zhang, Xiaomin Kang, Yuting Sun

**Affiliations:** ^1^ Guang’anmen Hospital, China Academy of Chinese Medical Sciences, Beijing, China; ^2^ Beijing University of Chinese Medicine, Beijing, China

**Keywords:** COVID-19, TCM (trad. Chinese medicine), recovery period, mechanism, long-COVID-19

## Abstract

The severe acute respiratory syndrome coronavirus - 2 (SARS - CoV - 2) was reported to cause the Wuhan outbreak of the corona virus disease 2019(COVID-19). To date, the COVID-19 has infected more than 600 million people gloabally. As a growing number of patients recover from acute infections and are discharged from hospitals, the proportion of patients in the recovery period is gradually increasing. Many of these individuals have been reported to experience multiple symptoms during the convalescence, such as fatigue, dyspnea and pain which are designated as “long-COVID”, “post-COVID syndrome” or “recovery sequelae. We searched for recent articles published in PubMed on COVID-19 convalescence and found that the pathogenesis of COVID-19 convalescence is not yet well recognized. It may be associated with incomplete recovery of immune system, parenchymal organ damage (liver or lung), coagulation abnormalities, “second hit” caused by viral infection, and Phenomenon of Cell Senescence-Associated Secretory Phenotype (SASP). Some drugs and psychological factors of patients also play a non-negligible role in it. We also found that the effect of traditional Chinese medicine (TCM) is effective in the treatment of the COVID-19 recovery phase, which can not only relieve the corresponding symptoms, but also improve the indicators and pulmonary fibrosis. Bufei Huoxue Capsule, as the only drug explicitly mentioned for COVID-19 recovery period, can exert strong rehabilitative effects on physiological activity in patients recovering from COVID-19. In addition, in previous studies, traditional Chinese medicine has been confirmed to have the ability to resist cytokine storms, as well as improve coagulation and myocardial damage, which makes it have potential therapeutic advantages in targeting the hyperimmune response, coagulation abnormalities and myocardial damage existing in the recovery period. In conclusion, the clinical symptoms of patients convalescing from COVID-19 are complex, and its pathogenesis has not been elucidated. traditional Chinese medicine, as a traditional treatment, its specific action and mechanism need to be confirmed by more studies, so that it can play a better role.

## 1 Introduction

The severe acute respiratory syndrome coronavirus - 2 (SARS - CoV - 2) (Supplementary Table S1 List of Abbreviations) was reported to cause the Wuhan outbreak of the corona virus disease 2019(COVID-19). As of 28 November 2022, there have been more than 600 million people infected with SARS - CoV - 2 (WHO. 2022), and this number is still increasing. Most patients infected with COVID-19 can be recovered and discharged after systemic treatment, and the period that patients discharged from rehabilitation subsequently is called recovery period. The discharge criteria are defined as: 1. body temperature returns to normal for 3 days; 2. respiratory symptoms are significantly improved; 3. pulmonary imaging shows that acute exudative lesions are significantly improved; 4. two consecutive negative nucleic acid tests of respiratory specimens (more than 24 h apart) (An X. et al., 2021). However, after mildly suspected COVID-19, a proportion of individuals experience a prolonged recovery and some of these patients also develop a wide variety of complications, such as viral myocarditis, thromboembolic complications and primary psychiatric phenomena (Salman et al., 2021). A multicenter prospective cohort study published in JAMA shows that some COVID-19 patients developed somatization symptoms, psychiatric symptoms and cognitive symptoms in a 1 year period after treated in the ICU, with frequently reported conditions including asthenia, joint stiffness, arthralgia, muscular weakness, and myalgia (Heesakkers et al., 2022).

The complexity of symptoms, lengthy disease course and large patient volumes make treatment during the recovery period of COVID-19 challenging. An editorial also pointed out that the management of long COVID is expected to become a global public health priority (Parums 2021). Therefore, we searched PubMed for the latest relevant literatures, giving due attention to the problems that exist in COVID-19 convalescence. The aim of this review is to summarize the symptoms and possible pathogenesis of COVID-19 convalescence and to clarify the potential of traditional Chinese medicine by studying the current application of Chinese medicine in COVID-19 recovery period to provide novel ideas for the treatment of COVID-19 convalescence.

## 2 Literature search terms, data base and methods

We searched the relevant literature (until 22 November 2022) mainly in PubMed database using medical subject headings (MeSH), and also supplemented the search in the China National Knowledge Infrastructure (CNKI) database and Wanfang database. The main English search terms were “Convalescence”“COVID-19”“TCM”, etc. For ease of reference, the MeSH terms and detailed search records are listed in Supplementary Presentation S1.

## 3 Common symptoms in the recovery period of COVID-19

**FIGURE 1:**
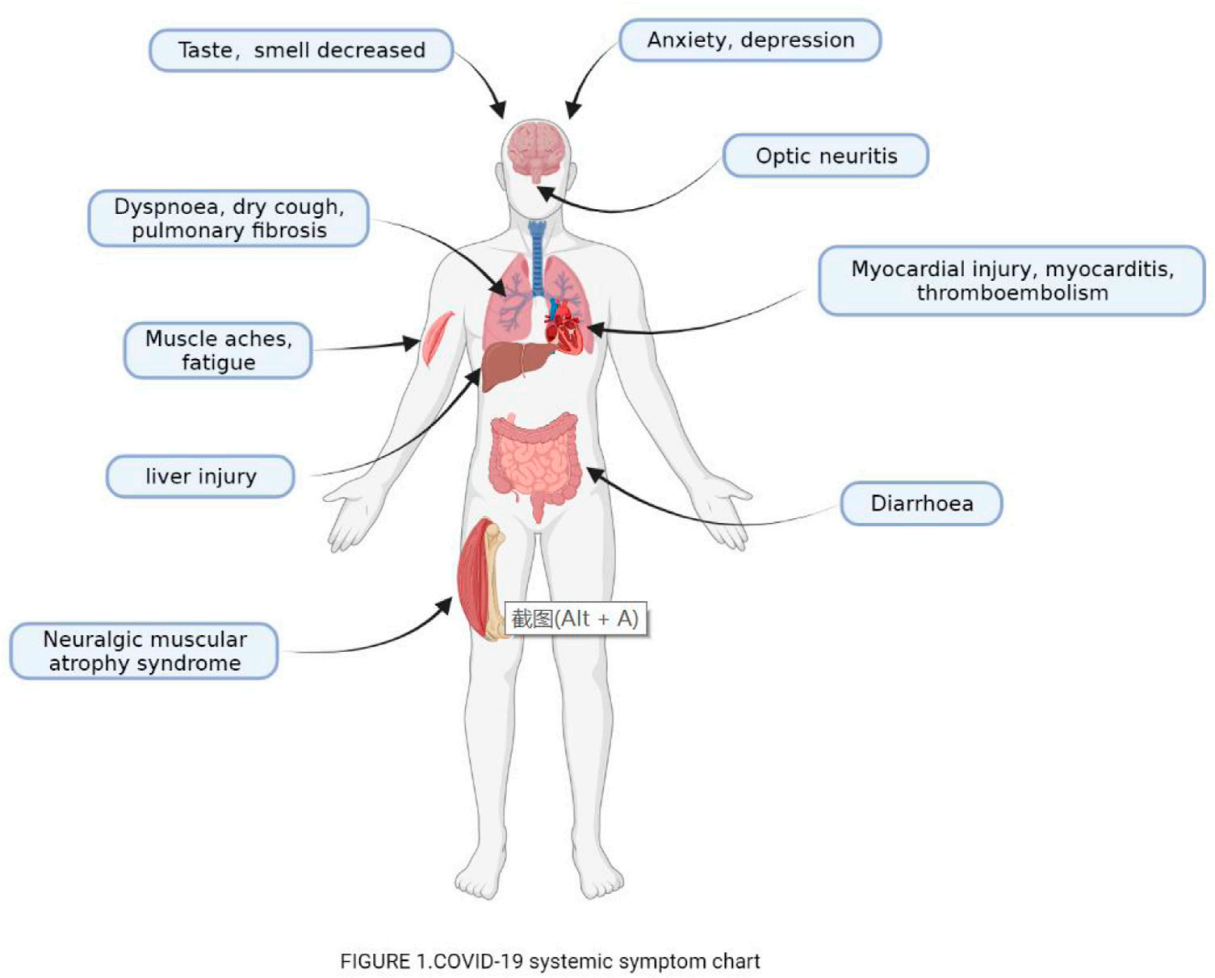
COVID-19 systemic symptom chart.

Numerous studies have shown that patients in recovery may develop a variety of sequelae or complications, such as fatigue, dyspnea, headache, myalgia, psychological symptoms (like memory dysfunction, sleep disorders, cognitive dysfunction, anxiety and depression or other affective disorders), taste and smell loss (Zhu et al., 2021). In addition, COVID-19 convalescent patients develop symptoms of various other systems (Figure 1), which together constitute a complex set of convalescent symptoms.

### 3.1 Effects on respiratory system

Most patients still have residual respiratory symptoms after rehabilitation, such as dry cough, fever, dyspnea, *etc.*; and persistent cough and dyspnea are the main symptoms in the recovery phase of severe COVID-19 (Miwa et al., 2021). It has been reported that approximately half of patients continue to have above persistent symptoms and lower lung function for 2 months after contracting COVID-19, which is common even in young SARS-CoV-2 recovered patients with fewer comorbidities (Trinkmann et al., 2021). Typically, patient self-reported walking time is commonly used to measure a patient’s lung function, and evidence suggests that self-reported walking time is still significantly reduced in most convalescent patients 6 months after the onset of symptoms (Delbressine et al., 2021). A 6-month follow-up study of 54 patients with COVID-19 discharged from the hospital showed that fatigue and exertional dyspnea would persist up to 6 months after hospital discharge (Wu et al., 2021). Continued cough and dyspnea are common symptoms in the recovery phase of PILS (posterior laryngotracheal stenosis) and severe COVID-19 (Miwa et al., 2021). A CT study of the lungs of patients in recovery found that fibrous streaks and ground glass opacities (GGO) are common CT signs in critically ill patients with COVID-19 pneumonia during the early recovery phase (Fang et al., 2020). There were 46% of COVID-19 patients (mainly severe or critical cases) with fibrotic changes on follow-up CT during the early recovery stage (Yang L. et al., 2020).

### 3.2 Effects on the cardiovascular system

There are many cardiovascular symptoms in the recovery period of COVID-19, such as thromboembolism, myocardial injury, myocarditis and so on. Research has shown that the risk of thrombosis in patients with COVID-19 not only exists in the acute phase, but also persist into the recovery phase. (Chan et al., 2021). Meanwhile, myocardial injury has also been reported during the recovery phase of COVID-19 (Ide et al., 2021) and elevated troponins during this period is not an unusual problem (Knight et al., 2020), which is equally present in patients without a previous history of cardiovascular disease and risk factors. Therefore, silent myocardial infarction is an important differential diagnosis to be considered for symptom aggravation during the recovery phase of COVID-19 (Tschöpe et al., 2021). In addition to the heart, there are microvascular abnormalities in the recovery period of COVID-19. As in a study on the effects of COVID-19 on human retinal microcirculation demonstrated that compared with healthy controls, the group of patients recovering from COVID-19 had larger areas of entire retinal no-flow and foveal ischemia (*p* > 0.05), and higher vascular density in the superficial parafoveal deep capillary plexus (*p* < 0.05) (Aydemir et al., 2021).

### 3.3 Effects on the neurological

One of the common symptoms in convalescent patients with COVID-19 is fatigue and muscle soreness, which may be neurologically related. Studies have shown that the most common symptom of long-term COVID-19 is “fatigue”, which is not necessarily triggered by exertion and cannot be relieved by rest (Alwan 2021). SARS-Cov-2 may contribute to various neuro-ophthalmologic manifestations, including optic nerve perineuritis (Ali et al., 2021). Anosmia and taste loss are also commonly seen in the recovery phase (Rao et al., 2021). A recent study in the Journal of the American Medical Association showed that 72% of patients recovering from COVID-19 had generalized muscle weakness, even more severe than in cancer patients (40%) as well as pain and difficulty with physical activity were also more common in these patients (Kuehn 2021). Furthermore, patients who experience COVID-19 are more likely to switch to chronic pain (Kemp et al., 2020).

### 3.4 Mood disorders

COVID-19 is associated with neuropsychiatric complications, the most common of which is anxiety, which may become a long-term complication (Uzunova et al., 2021). In addition to the acute infectious period, depression and anxiety are also prevalent in patients with COVID-19 rehabilitation. With the recovery from COVID-19, an increase in patients with psychosomatic diseases has been reported, as manifested by memory dysfunction, cognitive dysfunction, anxiety and depression or other affective disorders that may lead to mental decline (Sklinda et al., 2021). Patients who survive COVID-19 often present with anxiety and depression, with common symptoms including anticipatory anxiety about progression within physical therapy and occupational therapy (OT), anxiety about long-term recovery, depression associated with loss of function, and feelings of isolation. Many patients present with somatic anxiety symptoms, including spontaneous awakening, dizziness, and shortness of breath, which may result from multifactorial physical and psychological effects (Jaywant et al., 2021). A survey of 538 COVID-19 survivors revealed that 22.7% (n = 122) of these recovered patients had psychosocial symptoms, including depression, anxiety, irritability, and inferiority complex (Xiong et al., 2021).

### 3.5 Abnormal liver functions

Abnormal liver function is common in patients with COVID-19 (Gan et al., 2021) in China (Cai et al., 2020) and worldwide (Daugherty et al., 2021; Santana et al., 2022), as evidenced by elevated the alanine aminotransferase and aspartate aminotransferase (Santana et al., 2022). A United States-based retrospective cohort demonstrated that abnormal liver tests were commonly observed in hospitalized patients with COVID-19, both on admission (AST 66.9%, ALT 41.6%, ALP 13.5%, and TBIL 4.3%) and peak hospitalization (AST 83.4%, ALT 61.6%, ALP 22.7%, and TBIL 16.1%) and also during recovery. Abnormalities in liver tests may be associated with severe COVID-19 related causes, including ICU admission, mechanical ventilation, and death (Hundt et al., 2020). As we all know, some drugs may cause liver function damage in acute infectious diseases, such as Nonsteroidal Anti-inflammatory Drugs (NSAIDs). In COVID-19, drug-induced liver injury has also been reported, especially with lopinavir and ritonavir (Ali 2020). It has also been suggested that other drugs (such as tocilizumab) and other factors (such as ischemia) are associated with severe liver injury (Chew et al., 2021). In addition to external factors, acute coronavirus infection itself may contribute to this condition (Covid-19 Drugs, 2012). Several studies have found that increased severity of liver chemistry abnormalities (such as ALT-AST elevation、AST/ALT ratio >1 (Medetalibeyoglu et al., 2020)and high levels of aspartate aminotransferase [AST] and direct bilirubin [D-Bil](Ding et al., 2021)) on hospital admission predict early in-hospital mortality in COVID-19 patients (Satapathy et al., 2021). however, a cohort study considered that baseline liver test abnormalities were associated with an increased risk of ICU admission (OR 2.19 [95% CI 1.24–3.89], *p* = 0.007) but not with mortality (OR 0.84 [95% CI 0.49–1.41], *p* = 0.51) (Ponziani et al., 2020). However, some researchers disagree with the above views (Elmunzer et al., 2021).

Although there are different views, most physicians agree and value abnormal liver function in their patients, and the author also holds the same view. Especially for drug-induced liver injury (DILI), aggressive treatment is very meaningful for the rehabilitation of patients. For DILI, Roussel Uclaf Causality Assessment Method (RUCAM) remains the optimal method for diagnosis currently, including the updated RUCAM published in 2016 and the original RUCAM from 1993 (Teschke et al., 2022). Additionally, liver histologic findings may also contribute to the diagnosis (Ahmad et al., 2022).

### 3.6 Others

In addition, diarrhea. (An X. et al., 2021), massive pneumothorax (Nunna and Braun 2021), Guillain-Barré syndrome (Haidary et al., 2021), neuralgic muscular atrophy syndrome (Alvarez et al., 2021)and cerebrovascular complications (Cardoso et al., 2021) can likewise occur in the recovery period of the COVID-19, albeit at a low incidence.

## 4 Potential mechanisms of symptoms during the recovery period of COVID-19

**FIGURE 2:**
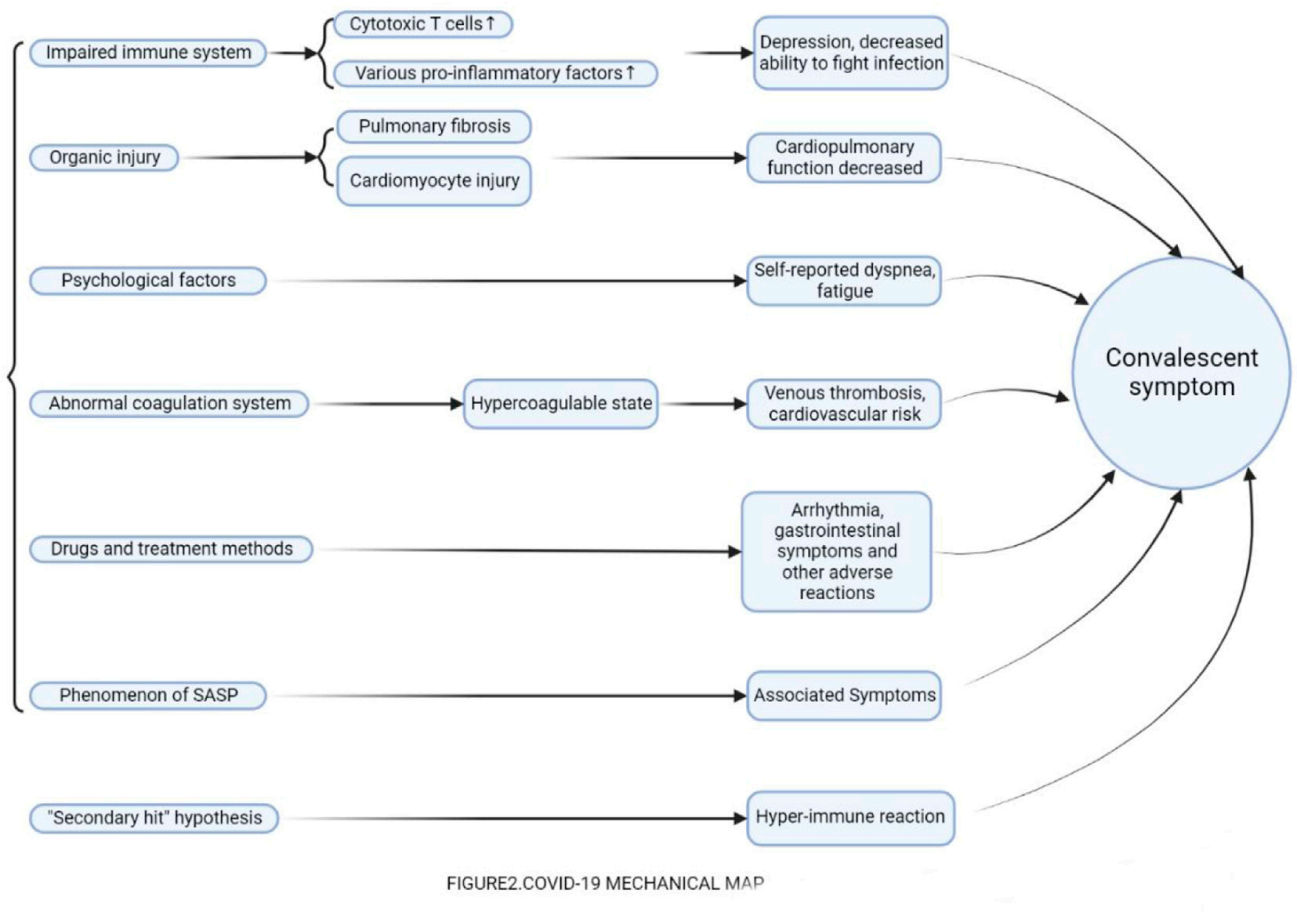
COVID-19 Mechanical map.

SARS-CoV-2 is an RNA virus similar to SARS coronavirus (SARS-CoV) and belongs to β coronavirus. SARS-CoV-2 infects respiratory mucosal epithelial cells by acting on angiotensin-converting enzyme II (ACE2) on the surface of human cells through the spike protein (S protein). ACE2 receptors are widely distributed in the lung, heart, ileum, kidney, bladder, and brain, which explains why COVID-19 is often involved in multisystem lesions, such as lung, spleen, hilar lymph nodes, bone marrow, heart, liver, gallbladder, kidney and other multiple organ or tissue damage manifestations. Currently, cytokine storm and immune dysfunction are the main pathogenic mechanisms in the acute phase of COVID-19, while in the convalescent phase, the pathogenesis of symptoms is rarely reported. Most studies suggest that it may be related to certain states left over from the acute phase (immune system and blood circulatory system), which leads to the symptoms described above. Some also suggest that these symptoms are simply motivated by the psychological effects of the patient. It is not negligible that certain treatments and medications used in the acute phase are indeed associated with certain symptoms. In addition, several hypotheses have been proposed that the symptoms during the recovery period may also be associated with the convalescence of COVID-19. Nevertheless, these possible mechanisms have not been recognized, and will be listed in detail below: (Figure 2).

### 4.1 Incomplete recovery of the compromised immune system

Studies have shown that SARS-CoV-2 disrupts the normal immune response, resulting in decreased lymphocyte count, lymphocyte activation and dysfunction, abnormal granulocytes and monocytes (abnormal number and ratio), increased cytokine levels and increased number of IgG antibodies (Yang L. et al., 2020). Lymphocyte consumption and cytokine storm further cause increased infections, multiple organ dysfunction and even failure, ultimately threatening the patient’s life.During the recovery stage, there are also reports of immune damage. A study of 78 convalescent patients infected with COVID-19 showed that those recovering patients maintained robust SARS-CoV-2–specific humoral and cellular responses for 1 year post-infection (Hou et al., 2021), as was similarly demonstrated in a prospective cohort study (Masiá et al., 2021). Based on a study at the Affiliated Hospital of Hubei University of Traditional Chinese Medicine, with the conversion of SARS-CoV-2 antibodies in the patients recovering from COVID-19, their immune status did not recover, and the dynamic changes of serum IgM and IgG antibodies closely related to the immune status of convalescent patients (Shen et al., 2021). Zhang et al. found that leukopenia and lymphopenia were common in critically ill patients discharged from the hospital (Zhang et al., 2020). Compared with healthy controls, COVID-19 recovered patients had higher levels of circulating endothelial cells (biomarkers of vascular injury) and a higher frequency of effector T cells. Several pro-inflammatory and activated T lymphocyte-associated cytokines persist from the acute infection to the recovery phase, which increases the possibility of cytotoxic effector cells targeting activated endothelial cells (Chioh et al., 2021). In another study, fifty patients were reviewed at a minimum of 6 weeks following acute COVID-19. It showed that abnormal T cell and monocyte populations may be significant in the context of persistent EC activation and hemostatic dysfunction during convalescence (Fogarty et al., 2022). In addition, a survey of 96 convalescent COVID-19 patients in Shenzhen, China, revealed that patients with self-reported depression showed increased immune responses, such as increased white blood cell and neutrophil counts, and changes in the ratio of neutrophils to lymphocytes (Yuan et al., 2020). Concluding these studies on immune parameters during the recovery period, as the virus is cleared from the human body and the patient enters the recovery period, the impaired immune function is not completely restored and the inflammatory factors that were elevated during the acute period may persist into the recovery period. This abnormal immune function may lead to various symptoms.

### 4.2 Parenchymal organ damage caused by viral infections remains

According to the latest Diagnosis and Treatment Plan for New Coronavirus Pneumonia, the eighth revision, the SARS-CoV-2 can cause damage to multiple organs, including: lung (pulmonary consolidation, thromboembolism, pulmonary interstitial fibrosis, *etc.*), spleen (spleen shrinkage, anemic infarction), heart (myocardial cell degeneration and necrosis, interstitial congestion and edema), liver (hepatocyte degeneration), kidney (renal interstitial congestion, tubular necrosis), brain tissue edema, and so on. These injuries persist even after the recovery period. Several studies have shown that fibrotic streaks and GGO are common CT signs in patients with severe COVID-19 pneumonia during the early recovery phase (Fang et al., 2020). Approximately 46% of patients with COVID-19 (mainly severe or critical cases) exhibit fibrosis on follow-up CT during the early recovery phase (Yang Z. L. et al., 2020). In addition to CT findings, pulmonary function was also abnormal in discharged patients. As a study of 186 patients recovering from COVID-19 confirmed 47% had restrictive respiratory dysfunction and that patients with persistent dyspnea had significantly lower FVC (forced vital capacity) (*p* = 0.03) (Cortés-Telles et al., 2021). Futhermore, a number of studies have reported myocardial injury, elevated troponin (Knight et al., 2020; Rivera-Morales et al., 2020), ST-segment elevation myocardial infarction (Wong et al., 2021) and other manifestations during the recovery period, which also occur in healthy patients without a history of cardiovascular disease and risk factors. In brief, substantial damage to organs (mainly the cardiopulmonary system) caused during the acute phase often cannot recover immediately with the clearance of the virus, which causes a series of symptoms, such as dyspnea and decreased exercise tolerance.

### 4.3 Persisted or prolonged abnormality of the blood coagulation system

It is well-known that patients with acute coronary pneumonia often accompanied by abnormalities in coagulation, such as a marked increase in platelet count (PLT), prothrombin time (PT), activated partial thromboplastin time (APTT), thrombin time (TT), fibrinogen (FIB) and plasma D-dimer, forming a hypercoagulable state. It has also been reported that this hypercoagulable state is not only present in the middle and later stages of COVID-19 disease, but can even persist into the recovery period (Chan et al., 2021). Through long-term follow-up studies, it has been found that deep venous thrombosis is not only present in the acute phase of COVID-19, but is also diagnosed in a significant number of patients in the weeks after discharge (Kirshblum et al., 2021). As Roberts et al. reported 9 cases of venous thromboembolism in 1877 post-discharge COVID-19-related patients within 42 days, an average of 4.8 cases per 1,000 (Roberts et al., 2020). Patients with COVID-19, especially those with pre-existing cardiovascular risk, may show signs of persistent vascular dysfunction even after recovery from COVID-19. Therefore, early hematologic assessment (thrombin generation, platelet activation tests, *etc.*) is necessary for patients entering the recovery phase, especially before complications such as thrombosis occur (Chioh et al., 2021). Abnormal coagulation function leads to thrombosis, which may cause a series of complications such as vascular embolism. In addition to thrombosis, there are other symptoms that may also be associated with coagulation. A research of 55 convalescent COVID-19 patients suggested that virus-induced endothelial cell alterations may play a key pathogenic role in the systemic manifestations of COVID-19, including pulmonary, vascular and neuropsychological manifestations (Moretta et al., 2022).

### 4.4 “Secondary hit” hypothesis due to SARS-cov-2 virus infection

It is now recognized that cytokine storm is the main factor resulting in severe COVID-19. Various cytokines (e.g., IL-1ß, IFN-γ, *etc.*) increase dramatically in the acute phase, leading to rapid deterioration high mortality in patients with COVID-19 (Kim et al., 2021). As the virus is gradually cleared and the patient is discharged into the recovery period, this excessive immune response gradually subsides, but this does not seem to represent the recovery of the immune system, and there seems to be some “hidden danger” remaining. A case report on cardiac surgery in a convalescent patient recovering from COVID-19 stated that the patient developed unexplained hypoxemia during surgery, and postoperatively developed a “storm course” involving bleeding, low cardiac output syndrome, and rhabdomyolysis of lower extremity muscles. Explaining this phenomenon, the authors stated that the virus causes a series of symptoms (first hit) during the acute phase of infection of the human body, while after the infection is eliminated, some hidden dangers are left behind, which may appear second hit, induced by situations such as surgical procedures, mechanical ventilation, blood transfusion, thrombosis, ischemia *etc.*, resulting in a series of serious symptoms (Damodaran et al., 2021). In our perspective, this second hit is like the immune system just finishing a fight (severe infection), but the immune system still maintains a state of high alert. This state may incorrectly perceives stimulI such as surgery as a viral attack, which leads to the second appearance of the course of the storm. The excessive immune response caused by this second hit may also be one of the reasons for the occurrence of symptoms in the recovery period of COVID-19.

### 4.5 Sequelae due to drugs and treatment modalities

As early as 2003, osteonecrosis of the femoral head caused by hormone use was reported, and in this COVID-19, there were also reports of drug-induced physical damage. Hydroxychloroquine and streptomycin are widely used in COVID-19, but some studies have shown that the combination of hydroxychloroquine and streptomycin can significantly prolong the QTc interval in COVID-19 patients thereby leading to life-threatening arrhythmias in the form of torsade de pointes (TdP) (Chorin et al., 2020). Moreover, the abuse of antiviral drugs also exists in clinical practice. For example, an analysis of acute drug treatment in 40 patients discharged from hospital with the New Coronavirus Pneumonia found that 67.5% of patients were clinically treated with more than three antiviral drugs, which was contrary to the “simultaneous use of more than three antiviral drugs is not recommended” specified in the “Diagnosis and Treatment Plan for New Coronavirus Pneumonia”, and that such overmedication increases gastrointestinal adverse effects (Ma et al., 2020). Besides drugs, some therapeutic measures for COVID-19s have also been reported to have some adverse effects. Mechanical ventilation is one of the essential therapeutic measures for patients in the acute phase, but it has been demonstrated that mechanical ventilation leads to a long-term decrease in exercise capacity (Saeki et al., 2021). Recovery lung function impairment may be related to admission radiological involvement (Truffaut et al., 2021). However, there are relatively few reports in this area, especially the lack of studies on the long-term outcome of COVID-19s dosing. At present, it only stays in the retrospective study stage, with small sample sizes and short study periods. The long-term effects of many drugs for the treatment of COVID-19 patients are not clear, which is also not available in high-quality RCT studies, and thus may influence the judgment of clinicians.

### 4.6 Abnormal psychological factors in patients

A large-scale investigative study recently published in JAMA Internal Medicine, a sub-journal of JAMA, pointed out that so-called long-term COVID-19 symptoms, may be caused more by psychological effects than by SARS-CoV-2 infection (Matta et al., 2022). In this study, a large proportion of patients who had already tested negative for COVID-19 antibodies believed they were infected with SARS-CoV-2 and reported long-term symptoms such as dyspnea and fatigue, and for this group of patients, the reported symptoms were considered to be caused by psychological effects rather than SARS-CoV-2. The results of this study suggest that patients with a long course of COVID-19 may also have symptoms related to the patient’s own psychological factors.

#### 4.7 Phenomenon of Cell Senescence-associated secretory phenotype (SASP)

Recently, a research team from Osaka University in Japan published a research paper entitled: SARS-CoV-2 infection triggers paracrine senescence and leads to a sustained senescence-associated inflammatory response in Nature Aging, a sub-journal of Nature. The research paper points out that infection of cells with SARS-CoV-2 induces the production of cellular senescence-associated secretory phenotype (SASP), which affects nearby uninfected cells to develop a senescence-like cell cycle arrest, which persists even after the SARS-CoV-2 is cleared. The presence of this phenotype, may be responsible for the long-term symptoms during the recovery phase of the COVID-19 (Tsuji et al., 2022).

## 5 The effect of Traditional Chinese Medicine

It is well-known that TCM has played a significant role in the fight against COVID-19 in China. Many herbal formulas have been shown to be efficacious and recommended for COVID-19, such as Jinhua Qinggan (JHQG) granules, Lianhua Qingwen (LHQW) capsules, Xuanfeibaidu (XFBD) granules, Huashibaidu (HSBD) and Xuebijing (XBJ) (Huang et al., 2021). There are also reports of cases in the recovery phase treated with TCM. A Prospective Cohort found that Chinese Medicine (CM) could improve pulmonary inflammation to promote early recovery (Li L. et al., 2021). This was also found in a retrospective analysis (An Y. W. et al., 2021). We conclude that the main effects of TCM against COVID-19 convalescence are as follows.

### 5.1 Relief of convalescent symptoms

At present, there are many studies on the relief of convalescent symptoms by traditional Chinese medicine. For example, studies on three syndromes and six drugs in the convalescent stage have shown that Jinshuibao Tablets and Shengmaiyin Oral Liquid significantly improve the cardiopulmonary function of patients. Shumian capsule and Xiangsha Liujunwan or Dangshen Oral Liquid can significantly improve patients’ sleep disorder and the digestive function respectively (An X. et al., 2021). In their experiments for the treatment of convalescent COVID-19, Chen Zhong observed that the method of cultivating earth and generating gold (Using the theory of traditional Chinese medicine, supplementing the lung by supplementing the spleen) could alleviate the symptoms of chest tightness, shortness of breath, fatigue, anorexia and distended abdomen in COVID-19 recovered patients (Chen 2021). Shi Suofang et al. used Futushengjin Kangfu Recipe to treat 89 patients with lung and spleen deficiency syndrome during the convalescence period of COVID-19, and the results showed that the recipe could effectively improve the symptoms of fatigue, shortness of breath, diarrhea with an effective rate as high as 94.38% (Shi et al., 2020). Yang Hongzhi et al. treated 60 patients with COVID-19 who had not cleared residual toxicity in the convalescence period with aromatic fire spray combined with basic rehabilitation therapy, and the results showed that after treatment, the symptom scores of cough, chest tightness, fatigue, sweating, muscle soreness, nasal congestion and sore throat were significantly lower in the treatment group than in the control group (*p* < 0.05) (Yang et al., 2021).

### 5.2 Improvement of organic organ injury

A network pharmacological study of convalescent COVID-19 prescription (CCP) on SARS-COV-2 infection-related pulmonary fibrosis showed that CCP could inhibit the expression of VEGF, TNF-α, IL-6, MMP9, and TGF-β1 through VEGF, Toll-like four receptor, MAPK, and TGF-β1 signaling pathways, which may be the reason why the CCP works as an anti-fibrotic (Jin et al., 2021). Network pharmacology has shown that Shenlingbaizhu powder can reduce pulmonary fibrosis by down-regulating VEGF expression (Lin et al., 2020). Furthermore, a number of studies, such as the intervention effect of Chinese herbal formula against pulmonary fibrosis (Bian et al., 2020), have shown that traditional Chinese medicine can improve pulmonary fibrosis in patients with COVID-19 through multiple targets and multiple ways.

### 5.3 Effects on immune function

Yin Yanyan et al. explored the mechanism of Buzhong Yiqi Decoction on the recovery period of COVID-19 based on network pharmacology, and found that Buzhong Yiqi Decoction could play a potential therapeutic role in the recovery period of COVID-19 by eliminating inflammation, reducing lung damage and regulating immunity through multiple targets and pathways (Yin et al., 2021). In the New Coronavirus Diagnosis and Treatment Plan (Eighth Revision), two prescriptions are recommended for lung and spleen deficiency syndrome and qi and yin deficiency syndrome in the recovery period, and studies have found that drugs, botanical drugs such as Astragalus membranaceus, Codonopsis pilosula, Atractylodes macrocephala, Nansha ginseng, and Ophiopogon japonicus, have the effects of increasing the number of leukocytes, stimulating the proliferation of T and B lymphocytes, improving phagocyte capacity, or balancing immunity (Geng and Xie 2021). Wang Bohan et al. ‘s network pharmacological analysis of Futushengjin Kangfu Fang showed that quercetin and luteolin contained in the formula could promote T lymphocyte differentiation, suppress pathological CD4 T lymphocytes, and exert a regulatory effect on immune function during the recovery period of COVID-19 (Wang et al., 2021).

## 6 Specific application of Traditional Chinese Medicine in COVID-19 recovery period

There are many studies affirming the role of TCM in the recovery phase of COVID-19, but most of them do not publish the specific prescription contents, and some prescriptions are “self-invention prescriptions” of TCM practitioners, which may hinder the promotion and application of TCM. As the only Chinese medicine explicitly mentioned for the treatment of COVID-19 convalescence, Bufei Huoxue (BFHX) capsules can relieve the symptoms of cough, palpitation and shortness of breath and other symptoms, consisting of three herbal ingredients from TCM: Astragalus membranaceus Bunge[Fabaceae, Astragalus L.], Paeoniae radix rubra [Ranunculaceae Juss, Paeonia L.], and Psoralea corylifolia L [Fabaceae, Psoralea Linn](The Official Product Information for Bufei Huoxue Capsule. 2022). Among its ingredients, Astragali radix has a broad-spectrum effects on the human body, enhancing the immune system, inproving tolerance against hypoxia, regulating organ function, and preventing microbial infections. Paeoniae radix rubra exerts its therapeutic effects by improving microcirculation, lowering the viscosity of serum and plasma, and clearing excessive “heat” and “cold” from the blood. Psoraleae fructus plays a role in strengthening myocardial function, dilating the coronary arteries, and increasing blood flow. In addition, according to some network-based pharmacological studies, BFHX exhibits therapeutic effects in those recovering from viral pneumonia by supressing inflammatory pathways, and it may exert strong rehabilitative effects on physiological activity in patients recovering from COVID-19, thereby attenuating symptoms of fatigue and improving exercise tolerance (Chen et al., 2022).

Although not much information was retrieved, it is fortunate that TCM treatment of COVID-19 convalescence seems to be gaining attention. A meta-analysis of TCM treatment in the COVID-19 recovery period is ongoing (Zhou et al., 2021). Clinically, the number of symptomatic patients in the convalescent period seeking TCM treatment is also increasing. We strongly believe in the role of TCM in this field and look forward to more of its relevant research findings in the future. Relevant TCM treatment datas are shown in Supplementary Tables S2, S3, S4

### 6.1 BFHX capsules product information

BFHX (Chinese medicine Z20030063, Guangdong Lei Yun Shang Pharmaceutical Co., Ltd. (Yunfu, Guangdong Province, China); batch number 022001; specifications: 0.35 g per capsule) was obtained in the form of hard capsules containing fine brown particles/powder that is slightly fragrant, sour, and bitter. BFHX is composed of three herbs: Psoralea corylifolia L (Buguzhi) (40%), Astragalus membranaceus Bunge(Huangqi) (40%), and Paeoniae radix rubra (Chishao) (20%). The drug quality standards conform to the regulations of the Chinese Pharmacopoeia.

### 6.2 Pharmacokinetic study and mechanism study of BFHX capsule

BFHX contains eight main bioactive compounds (psoralen, isopsoralen, neobabaisoflavone, corylin, bavachin, astragaloside IV, ononin and formononetin), In an animal trial, men et al. obtained pharmacokinetic profiles (such as mean plasma concentration–time 、bioavailability 、pharmacokinetic parameters, *etc.*) of eight active ingredients by a sensitive and reliable ultra-highperformance liquid chromatography-mass spectrometrymethod. In terms of mechanism, in a mouse model of PM2.5-induced inflammation established with intranasal instillation of PM2.5 suspension, BFHX significantly reduced pathological response and inflammatory mediators including IL-4, IL-6, IL-10, IL-8, TNF-α, and IL-1β. BFHX also reduced keratinocyte growth factor (KGF), secretory immunoglobulin A (sIgA), and collagen fibers deposition in lung and improved lung function.

## 7 Potential advantages of traditional Chinese medicine in the COVID-19 recovery period

Although there is currently no evidence that COVID-19 requires treatment during the recovery phase, experts have called for research to identify appropriate therapies such as vaccines and antiviral drugs to prevent sequelae (Kalimuddin et al., 2022). Studies have also analyzed pharmacological treatment in the recovery phase and found that it is mainly symptomatic supportive therapy, while the use of antibiotics and antivirals is ineffective (Liu et al., 2020). We consider that it may be related to the gradual clearance of the virus as the nucleic acid test results turn negative during the recovery period.

The advantage of TCM in the treatment of COVID-19 convalescence is that it can not only improve symptoms, but also regulate its complex pathogenic mechanism in a holistic way. First of all, traditional Chinese medicine have the effect of improving immune function. A variety of traditional Chinese medicine ingredients specified in the diagnosis and treatment plan can play a role in improving the damaged immune system, such as stimulating lymphocyte proliferation, inhibiting pathological CD4 T cell proliferation, enhancing phagocytic ability and balancing immunity. Secondly, in terms of organic injury caused by pneumonia, network pharmacology studies have shown that traditional Chinese medicine can improve pulmonary fibrosis in the recovery period of COVID-19. In addition, traditional Chinese medicine can also improve coagulation function and myocardial injury, although there is no study related to its application in the recovery period of COVID-19, Yan et al. found that Chinese herbal medicine (Gualou Xiebai Decoction) significantly protected the myocardium from I/R injury, and reduced CK, CK-MB, LDH, cTnI, cTnT and IL-6 levels, improved cardiac function, and reduced myocardial injury in hyperlipidemic rats (Yan et al., 2018). In addition, traditional Chinese medicine is also helpful for hypercoagulable state, Yu et al. discovered that Xihuang pill combined with chemotherapy could prolong the thromboplastin time in patients with advanced colon cancer, thereby improving the hypercoagulable state of patients (Yu and An 2017). Targeting the possible “second hit” in convalescent patients of COVID-19, traditional Chinese medicine could be of benefit. Li et al. found that Qingfei Paidu and Xuanfei Baidu Decoction has a powerful function of reducing the expression of pro-inflammatory cytokines (interleukin-6, tumor necrosis factor-α), inhibiting the activation of NF-κB signaling pathway, and attenuating the exocytic activity of THP-1-derived macrophages (Li L. et al., 2021). Through a large-scale transcriptional study, Dai et al. found that Chinese botanical drugs have the power of inhibiting COVID-19-related cytokine storm (Dai et al., 2021).

Based on the above findings, it can be evidenced that TCM has great potential in the convalescent treatment of COVID-19. In addition, because of its simplicity and efficiency, TCM also has a unique advantage over western medicine when dealing with such a multi-stage, complex and variable symptoms in the recovery period of COVID-19. A slight deficiency is that the exact mechanism of traditional Chinese medicine in the treatment of COVID-19 recovery period is still unclear. Most studies are based on the predictions from network pharmacology, rather than objective clinical trials. Furthermore, most clinical trials only focus on the symptom level, without exploring specific mechanisms and focusing on objective indicators. In terms of organic injury, most of them only focus on pulmonary fibrosis, while other studies such as myocardial injury and cerebrovascular complications in the recovery period are lacking. Therefore, there is still a need to actively carry out targeted animal experiments, cell experiments and clinical trials to further elucidate the mechanism of action of traditional Chinese medicine on the recovery period of COVID-19.

## 8 Discussion

The recovery period of COVID-19 has complex multi-system symptoms, which can cause a variety of abnormalities in the circulatory system, respiratory system, digestive system, nervous system, and psychological disorders. The pathogenesis is not yet recognized which possible association with incomplete immune system recovery, parenchymal organ damage (liver or lung), coagulation abnormalities, “second hit” caused by viral infections, and Phenomenon of Cell Senescence-Associated Secretory Phenotype (SASP). There is no definite treatment for COVID-19 in the recovery period, hence symptomatic treatment is mostly used in clinical practice. Traditional Chinese medicine can be effective in the treatment of COVID-19 recovery period to alleviate clinical symptoms, regulate immunity, and improve pulmonary fibrosis. Nowadays, traditional Chinese medicines represented by Bufei Huoxue Capsules have been widely applied to COVID-19 convalescent patients, attaining good efficacy. Previous studies have confirmed that traditional Chinese medicine has the ability to alleviate cytokine storms and improve coagulation and myocardial damage, which offers potential therapeutic advantages in targeting the hyperimmune response, coagulation abnormalities and myocardial damage existing in the recovery period. These evidences all suggest the therapeutic significance of TCM in the convalescent phase of COVID-19.

At present, the main problems in the convalescent treatment of COVID-19 are long duration, many and complex symptoms, and unknown pathogenic mechanisms. Traditional Chinese medicine treatment also faces many challenges, such as too small sample size for clinical studies and less high-quality studies. In the future, we look forward to TCM carrying out the following aspects of research, so that it can be better applied to the COVID-19 recovery period. First, clinical trials of Chinese patent medicines targeting specific symptoms during the recovery phase of COVID-19. TCM has a long history and rich knowledge of botanical drugs, and promoting the marketing of Chinese patent medicines through clinical trials will facilitate patients in the recovery period of COVID-19. Secondly, carry out the study on the mechanism of traditional Chinese medicine in the recovery period of COVID-19. Animal experiments and cell experiments can be carried out to clarify its pathogenic mechanism. In China, TCM played an outstanding role in the COVID-19 epidemic, and we also look forward to its demeanor in convalescent treatment.

In summary, TCM has played a beneficial role in the convalescent treatment of COVID-19, and its specific role and mechanism need to be confirmed by more clinical trials and mechanistic studies. With the development of these studies, TCM is bound to play a greater role in the treatment of COVID-19 convalescence.

## Abbreviations

SARS - CoV-2, severe acute respiratory syndrome coronavirus - 2; COVID-19, corona virus disease 2019; TCM, traditional Chinese medicine; SASP, Senescence-Associated Secretory Phenotype; CNKI, China National Knowledge Infrastructure; GGO, ground glass opacities; PT, physical therapy; OT, occupational therapy; AST, Aspartate Transaminase; D-BiL, direct bilirubin; ALT, alanine transaminase; NSAIDs, Nonsteroidal Anti-inflammatory Drugs; ACE2, angiotensin-converting enzyme II; PLT, platelet count; PT, prothrombin time; APTT, activated partial thromboplastin time; TT, thrombin time; FIB, fibrinogen; TdP, torsade de pointes; JHQG, Jinhua Qinggan granules; LHQW, Lianhua Qingwen capsules; XFBD, Xuanfeibaidu granules; HSBD, Huashibaidu; XBJ, Xuebijing; CM, Chinese Medicine; CCP, convalescent COVID-19 prescription; BFHX, Bufei Huoxue capsules; KGF, keratinocyte growth factor; sIgA; secretory immunoglobulin A.
