# Supplementary material for: The pathological mechanism of the COVID-19 convalescence and its treatment with traditional Chinese medicine
Source: Front Pharmacol. 2023 Jan 10;13:1054312. doi: 10.3389/fphar.2022.1054312 (PMC9872123; doi:10.3389/fphar.2022.1054312)
Supplement: Supplementary file 1 [file Table1.docx]

| Abbreviation | DETAILED NAME |
| --- | --- |
| SARS - CoV - 2 | severe acute respiratory syndrome coronavirus - 2 |
| COVID - 19 | corona virus disease 2019 |
| TCM | traditional Chinese medicine |
| SASP | Senescence-Associated Secretory Phenotype |
| CNKI | China National Knowledge Infrastructure |
| GGO | ground glass opacities |
| PT | physical therapy |
| OT | occupational therapy |
| AST | Aspartate Transaminase |
| D-BiL | direct bilirubin |
| ALT | alanine transaminase |
| NSAIDs | Nonsteroidal Anti-inflammatory Drugs |
| ACE2 | angiotensin-converting enzyme II |
| PLT | platelet count |
| PT | prothrombin time |
| APTT | activated partial thromboplastin time |
| TT | thrombin time |
| FIB | fibrinogen |
| TdP | torsade de pointes |
| JHQG | Jinhua Qinggan granules |
| LHQW | Lianhua Qingwen capsules |
| XFBD | Xuanfeibaidu granules |
| HSBD | Huashibaidu |
| XBJ | Xuebijing |
| CM | Chinese Medicine |
| CCP | convalescent COVID-19 prescription |
| BFHX | Bufei Huoxue capsules |
| KGF | keratinocyte growth factor |
| sIgA | secretory immunoglobulin A |

List Of Abbreviations
