# Supplementary material for: The pathological mechanism of the COVID-19 convalescence and its treatment with traditional Chinese medicine
Source: Front Pharmacol. 2023 Jan 10;13:1054312. doi: 10.3389/fphar.2022.1054312 (PMC9872123; doi:10.3389/fphar.2022.1054312)
Supplement: Supplementary file 3 [file Table2.docx]

Comparison of patient characteristics at baseline.

| **Term** | **BFHX (N = 64)** | **Placebo (N = 65)** | **P-value [a](https://www.ncbi.nlm.nih.gov/pmc/articles/PMC8575540/table/tbl1/?report=objectonly#tbl1fna)** |
| --- | --- | --- | --- |
| Male (%) | 31 (48.44) | 29 (44.62) | 0.7254 |
| Age (years, x¯±s) | 54.16 ± 12.11 | 52.51 ± 12.31 | 0.4448 |
| Age group (years, %) |  |  | 0.7810 |
| 18–40 | 6 (9.38) | 8 (12.31) |  |
| 41–64 | 47 (73.44) | 44 (67.69) |  |
| 65–75 | 8 (12.50) | 11 (16.92) |  |
| >75 | 3 (4.69) | 2 (3.08) |  |
| Height (cmx¯±s) | 164.80 ± 7.21 | 163.94 ± 7.65 | 0.5163 |
| Weight (kgx¯±s) | 68.08 ± 11.78 | 65.92 ± 9.80 | 0.2603 |
| BMI (kg/m2x¯±s) | 24.995 ± 3.536 | 24.474 ± 2.894 | 0.3607 |
| Han nationality (%) | 64 (100.00) | 65 (100.00) | – |
| Manual labour (%) | 12 (18.75) | 9 (13.85) | 0.4835 |
| Marriage |  |  | 0.3800 |
| Married | 58 (90.63) | 62 (95.38) |  |
| Unmarried | 5 (7.81) | 3 (4.62) |  |
| Other | 1 (1.56) | 0 (0.00) |  |
| Severe/critical patients (%) | 13 (20.31) | 7 (10.77) | 0.1516 |
| Time from confirmation to randomisation (day) | 131.2 ± 14.0 | 130.7 ± 14.6 | 0.8254 |
| Time from discharge to randomisation (day) | 94.3 ± 17.0 | 94.3 ± 20.5 | 0.9845 |
| Comorbidities (N, %) | | | |
| Rheumatic diseases | 1 (1.56) | 0 (0.00) | 0.4961 |
| Respiratory diseases | 2 (3.13) | 1 (1.54) | 0.6191 |
| Urinary system diseases | 1 (1.56) | 1 (1.54) | 1.0000 |
| Endocrine and metabolic system diseases | 11 (17.19) | 10 (15.38) | 0.8155 |
| Nervous system diseases | 1 (1.56) | 0 (0.00) | 0.4961 |
| Digestive system diseases | 1 (1.56) | 6 (9.23) | 0.1148 |
| Cardiovascular diseases | 12 (18.75) | 13 (20.00) | 1.0000 |
| Ophthalmic Diseases | 1 (1.56) | 0 (0.00) | 0.4961 |
| Classification of concomitant medication (N, %) | | | |
| Digestive and metabolic system | 10 (15.63) | 5 (7.69) | 0.1807 |
| Cardiovascular system | 9 (14.06) | 10 (15.38) | 1.0000 |
| Chinese medicine/Chinese patent medicine | 3 (4.69) | 1 (1.54) | 0.3652 |
| Blood system | 2 (3.13) | 5 (7.69) | 0.4401 |
| Endocrine system | 1 (1.56) | 1 (1.54) | 1.0000 |
| Respiratory system | 1 (1.56) | 0 (0.00) | 0.4961 |
| Motor system | 1 (1.56) | 0 (0.00) | 0.4961 |
| Nervous system | 1 (1.56) | 0 (0.00) | 0.4961 |
| Antibiotics | 1 (1.56) | 0 (0.00) | 0.4961 |
| Combined | 1 (1.56) | 0 (0.00) | 0.4961 |

aP-values were calculated for continuous outcomes with t-tests for the change from baseline to the last visit after three months of treatment; Fisher’ s exact test was performed for categorical outcomes.

Changes in primary and secondary indicators from baseline to after three months of treatment [a](https://www.ncbi.nlm.nih.gov/pmc/articles/PMC8575540/table/tbl2/?report=objectonly#tbl2fna)

| **Indicator** | **BFHX (N = 64) [b](https://www.ncbi.nlm.nih.gov/pmc/articles/PMC8575540/table/tbl2/?report=objectonly#tbl2fnb)** | | | | **Placebo (N = 65) [b](https://www.ncbi.nlm.nih.gov/pmc/articles/PMC8575540/table/tbl2/?report=objectonly#tbl2fnb)** | | | | **Least Squares Means Differences (95% CI)** | **P-value[b](https://www.ncbi.nlm.nih.gov/pmc/articles/PMC8575540/table/tbl2/?report=objectonly#tbl2fnb)** |
| --- | --- | --- | --- | --- | --- | --- | --- | --- | --- | --- |
|  | **No. of patients[c](https://www.ncbi.nlm.nih.gov/pmc/articles/PMC8575540/table/tbl2/?report=objectonly#tbl2fnc)** | **Baseline** | **After 3 months of treatment** | **Change** | **No. of patients[c](https://www.ncbi.nlm.nih.gov/pmc/articles/PMC8575540/table/tbl2/?report=objectonly#tbl2fnc)** | **Baseline** | **3 months after treatment** | **Change** |  |  |
| Primary indicator | | | | | | | | | | |
| Chest CT Indexes | | | | | | | | | | |
| Volume of total lung lesions (cm³) | 53 | 31.5 ± 120.5 | 14.3 ± 32.1 | -19.2 ± 96.4 | 51 | 15.1 ± 39.4 | 29.2 ± 75.4 | 15.1 ± 47.5 | -34.0 (-63.5 to -4.6) | 0.0243 |
| Volume of the ground-glass opacities (cm³) | 53 | 29.3 ± 115.4 | 13.2 ± 30.6 | -18.3 ± 92.0 | 51 | 14.2 ± 38.2 | 23.9 ± 71.4 | 10.5 ± 42.4 | -32.6 (-60.7 to -4.5) | 0.0444 |
| Volume of the consolidations (cm³) | 53 | 1.8 ± 34.4 | 1.1 ± 2.3 | -0.9 ± 4.7 | 51 | 1.0 ± 1.6 | 2.0 ± 4.1 | 1.1 ± 3.4 | -1.4 (-2.6 to -0.1) | 0.0188 |
| 6-Min Walk Distance (m) | 58 | 427.3 ± 73.6 | 475.6 ± 63.7 | 45.3 ± 62.4 | 60 | 435.0 ± 73.3 | 445.5 ± 69.1 | 10.1 ± 59.4 | 34.2 (11.7–56.8) | 0.0022 |
| Secondary indicator | | | | | | | | | | |
| Fatigue Assessment Inventory | 58 | 119.1 ± 26.2 | 85.5 ± 27.6 | -31.2 ± 27.0 | 60 | 112.9 ± 31.6 | 100.4 ± 25.7 | -12.5 ± 36.1 | -17.8 (-29.5 to -6.2) | 0.0019 |
| Total SGRQ | 58 | 16.0 ± 12.1 | 3.2 ± 2.9 | -12.0 ± 10.9 | 60 | 14.0 ± 10.1 | 4.5 ± 4.2 | -9.1 ± 8.6 | -2.4 (-5.8 to 1.0) | 0.1148 |
| Part 1 SGRQ | 58 | 4.0 ± 4.3 | 0.5 ± 0.9 | -3.2 ± 4.0 | 60 | 3.8 ± 4.0 | 0.5 ± 1.1 | -3.4 ± 4.1 | 0.3 (-1.2 to 1.7) | 0.8310 |
| Part 2 SGRQ | 58 | 9.3 ± 8.0 | 1.4 ± 2.4 | -7.5 ± 7.1 | 60 | 7.5 ± 6.5 | 2.3 ± 3.3 | -4.9 ± 4.9 | -2.2 (-4.4 to -0.1) | 0.0234 |
| Borg Dyspnea score | 58 | 2.1 ± 1.3 | 0.7 ± 1.2 | -1.3 ± 0.9 | 60 | 2.1 ± 1.2 | 0.9 ± 1.4 | -1.2 ± 1.3 | -0.1 (-0.5 to 0.2) | 0.4801 |
| Chinese medicine symptom complex score | 58 | 4.3 ± 2.5 | 1.1 ± 1.7 | -3.1 ± 2.6 | 60 | 4.5 ± 2.8 | 0.9 ± 1.2 | -3.4 ± 2.4 | 0.4 (-0.4 to 1.3) | 0.4723 |

‡ P-values were calculated for continuous outcomes with t-tests for the change from baseline to the last visit after three months of treatment; Fisher’ s exact test was performed for categorical outcomes.

aData are presented as the means ± standard deviations. The changes from baseline to the end of three months of treatment were arithmetic. N lrb% is the number of patients and percentage. The least squares mean difference was calculated by analysing the generalised linear regression model with site as a confounder.

bTotal patients are allocated to the intention-to-treat population.

cNo. of patients observed at end of three months of treatment.
