# Supplementary material for: The pathological mechanism of the COVID-19 convalescence and its treatment with traditional Chinese medicine
Source: Front Pharmacol. 2023 Jan 10;13:1054312. doi: 10.3389/fphar.2022.1054312 (PMC9872123; doi:10.3389/fphar.2022.1054312)
Supplement: Supplementary file 4 [file Table3.docx]

Influences of TCM treatment on immune factors in COVID-19 convalescents

| **Indices (unit)** | **Reference range** | **Non-treatment (n = 425)** | **TCM treatment (n = 143)** | **P value** |
| --- | --- | --- | --- | --- |
| WBC count (109/L) | 3.5-9.5 | 6.01±1.38 | 5.68±1.05**↓ | 0.0078 |
| LYM count (109/L) | 1.1-3.2 | 1.84±0.55 | 1.73±0.43*↓ | 0.0234 |
| MON count (109/L) | 0.1-0.6 | 0.49±0.17 | 0.44±0.12**↓ | 0.0033 |
| NEUT count (109/L) | 1.8-6.3 | 3.52±1.17 | 3.32±0.90 | 0.0734 |
| EOS count (109/L) | 0.02-0.52 | 0.13±0.09 | 0.15±0.14 | 0.1286 |
| BASO count (109/L) | 0-0.06 | 0.04±0.02 | 0.04±0.01 | 0.8098 |
| IL-6 (pg/mL) | <7 | 18.31±54.06 | 4.47±17.53*↓ | 0.0113 |
| Hs-CRP (mg/dL) | 0-0.5 | 0.42±0.69 | 0.58±0.64 | 0.080 |
| SAA (mg/L) | 0-10 | 6.20±5.51 | 6.06±8.82 | 0.8293 |
| PCT1 (ng/mL) | 0-0.5 | 0.04±0.04 | 0.02±0.03*↓ | 0.0372 |

Continuous variables were analyzed using one-way ANOVA (Tamhane's T2) test. *P<0.05, **P<0.01 vs non-treatment group. The symbols ↓ and ↑ represented significantly higher or lower.

Comparison of blood biochemistry indices between different treatments in COVID-19 convalescents

| **Indices** | **Reference range** | **Non-treatment(n = 425)** | **TCM treatment(n = 143)** | **P value** |
| --- | --- | --- | --- | --- |
| ****Liver function**** |  |  |  |  |
| GLB (g/L) | 20-40 | 26.81±4.66 | 26.61±3.83 | 0.6427 |
| A/G | 1.5-2.5 | 1.75±0.31 | 1.79±0.30 | 0.1587 |
| AST/ALT | 0.5-1.5 | 1.42±0.64 | 1.36±0.63 | 0.3558 |
| GGT (U/L) | 7-60 | 35.10±33.06 | 27.31±18.13**↓ | 0.0088 |
| ALT (U/L) | 7-50 | 20.70±19.87 | 20.55±16.36 | 0.9398 |
| TBA (µmol/L) | 0-10 | 7.09±6.74 | 7.82±4.54 | 0.2386 |
| PA (g/L) | 0.2-0.4 | 0.29±0.06 | 0.35±0.62*↑ | 0.0492 |
| ALB (g/L) | 35-52 | 45.68±4.11 | 46.66±2.85**↑ | 0.0098 |
| TP (g/L) | 65-85 | 72.50±4.71 | 73.27±3.89 | 0.0818 |
| ****Kidney function**** | | | | |
| CysC (mg/L) | 0-1.5 | 0.96±0.22 | 0.96±0.15 | 0.8490 |
| UA (µmol/L) | 119-416.5 | 315.30±78.2 | 316.80±76.43 | 0.8452 |
| Urea (mmol/L) | 2.78-8.07 | 4.39±1.40 | 4.35±1.05 | 0.7672 |
| Cr (µmol/L) | 45-104 | 58.49±17.54 | 58.60±16.30 | 0.9451 |
| CO2CP (mmol/L) | 21-31 | 24.54±2.79 | 24.79±2.38 | 0.3435 |
| β2-MG (mg/L) | 0.8-2.2 | 1.768±0.68 | 1.69±0.47 | 0.2247 |
| ****Blood lipid level**** | | | | |
| ApoAⅠ/ApoB | 0.8-2.2 | 1.80±0.58 | 1.70±0.54 | 0.0799 |
| LP(a) (mg/dL) | 0-30 | 20.76±22.76 | 19.02±21.99 | 0.4322 |
| ApoB (g/L) | 0.60-1.33 | 0.85±0.23 | 0.93±0.26***↑ | 0.0009 |
| ApoAⅠ (g/L) | 1.04-2.25 | 1.44±0.28 | 1.46±0.23 | 0.4438 |
| TC (mmol/L) | 0-5.2 | 4.98±1.15 | 5.18±1.18 | 0.0749 |
| TG (mmol/L) | 0-2.6 | 2.42±2.02 | 2.59±1.47 | 0.3644 |
| HDL-C (mmol/L) | 1-1.55 | 1.20±0.35 | 1.17±0.29 | 0.3449 |
| LDL-C (mmol/L) | 1.90-3.10 | 2.91±0.93 | 3.13±1.05 *↑ | 0.0194 |
| ****Myocardial enzyme spectrums**** | | | | |
| α-HBDH (U/L) | 78-182 | 125.46±25.51 | 124.07±26.12 | 0.5842 |
| LDH (U/L) | 135-214 | 174.70±37.35 | 166.32±32.89*↓ | 0.0195 |
| CK-MB (U/L) | 0-25 | 6.14±4.00 | 5.83±3.41 | 0.4233 |
| CK (U/L) | 20-200 | 26.26±39.13 | 20.18±13.97 | 0.0764 |
| AST (U/L) | 15-40 | 22.45±12.93 | 22.14±9.74 | 0.7984 |

Continuous variables were analyzed using one-way ANOVA (Tamhane's T2) test. *P<0.05, **P<0.01, ***P<0.001 vs non-treatment group. The symbols ↓ and ↑ represented significantly higher or lower.

Comparison of blood routine indices between different treatments in COVID-19 convalescents

| **Indices (unit)** | **Reference range** | **Non-treatment (n = 425)** | **TCM treatment (n = 143)** | **P value** |
| --- | --- | --- | --- | --- |
| ****Platelet system**** |  |  |  |  |
| PLT (109/L) | 125-350 | 191.36±105.01 | 238.62±56.99***↑ | 0.0000 |
| PCT2 (%) | 0.18-0.39 | 0.21±0.10 | 0.25±0.05***↑ | 0.0000 |
| MPV (fL) | 6.5-12 | 10.92±1.10 | 10.72±0.94 | 0.0590 |
| P-LCR (%) | 17.5-42.3 | 32.07±8.65 | 30.60±7.70 | 0.0747 |
| PDW | 15-17 | 12.88±2.72 | 12.73±2.16 | 0.5506 |
| ****Erythrocyte system**** |  |  |  |  |
| RBC (1012/L) | 3.8-5.1 | 4.47±0.58 | 4.66±0.45***↑ | 0.0003 |
| MCV (fL) | 82-100 | 93.04±5.75 | 91.78±3.88*↓ | 0.0145 |
| RDW-SD (fL) | 38.2-49.2 | 45.58±6.82 | 43.84±4.21**↓ | 0.0042 |
| RDW-CV (%) | 12.1-14.3 | 13. 41±1.56 | 13.04±1.12**↓ | 0.0089 |
| HCT (%) | 35-45 | 41.36±4.28 | 42.70±3.57**↑ | 0.0008 |
| ****Hemoglobin parameter**** |  |  |  |  |
| HGB (g/L) | 115-150 | 132.97±15.77 | 139.64±14.41***↑ | 0.0000 |
| MCH (pg) | 27-34 | 29.86±1.80 | 29.97±1.62 | 0.5112 |
| MCHC (g/L) | 316-354 | 321.20±12.93 | 326.58±10.27***↑ | 0.0000 |

Continuous variables were analyzed using one-way ANOVA (Tamhane's T2) test. *P<0.05, **P<0.01, ***P<0.001 vs non-treatment group. The symbols ↓ and ↑ represented significantly higher or lower.
