# Supplementary material for: The pathological mechanism of the COVID-19 convalescence and its treatment with traditional Chinese medicine
Source: Front Pharmacol. 2023 Jan 10;13:1054312. doi: 10.3389/fphar.2022.1054312 (PMC9872123; doi:10.3389/fphar.2022.1054312)
Supplement: Supplementary file 5 [file Table4.docx]

| **Authors** | **Year** | **Journals** | **Study Design** | **Number of patients treated** | **TCM formulas** | **Patients’ symptoms** | **Control group** | **Outcome** |
| --- | --- | --- | --- | --- | --- | --- | --- | --- |
| **^&^[Yuqin Chen](https://pubmed.ncbi.nlm.nih.gov/?term=Chen+Y&cauthor_id=34763045) et al.** | **2022** | **J Ethnopharmacol** | **randomised controlled trial** | **66** | **^#^Bufei Huoxue capsules** | **fatigue, residual lung damage, and impaired exercise tolerance** | **Placebo n=65** | **improvements in exercise tolerance;**  **imaging manifestations on chest computed tomography (CT).** |
| **^&^[Ya-Wen An](https://pubmed.ncbi.nlm.nih.gov/?term=An+YW&cauthor_id=33437199) et al.** | **2021** | **Int J Med Sci** | **retrospectively analyzed** | **191** | **Shen ling bai zhu san and Xiao chai hu tang** | **psychiatric symptoms, gastrointestinal reaction and low immunity** | **no** | **white blood cell count；serum interleukin-6 and procalcitonin；**  **Serum γ-glutamyl transpeptidase et al.** |
| **[Li Li](https://pubmed.ncbi.nlm.nih.gov/?term=Li+L&cauthor_id=33534076) et al.** | **2021** | **Chin J Integr Med .** | **Prospective Cohort and Nested Case-Control Study** | **64** | ***Chinese Medicine** | **fatigue, sputum, cough, dry throat, thirst, and upset** | **Placebo n=32** | **Improvement rate of clinical symptoms;**  **Evolution of CM syndrome;**  **Complete lung inflammation absorption rate** |

**TCM treatment data**

**^#^Bufei Huoxue Capsules including Astragali radix, Paeoniae radix rubra, and Psoraleae fructus**

***Chinese medicine includes two: (1) for the pathogen residue syndrome: Salvia miltiorrhiza 15 g, prepared Fructus hordei germinates 30 g, Fructus hordei germinates 30 g, Codonopsis pilosula 15 g, Adenophora stricta 15 g, Peach kernel 6 g, Melon burdock 20 g, Magnolia offifi cinalis 10 g, Radix Reed 30 g, and Herba patriniae 30 g were decocted into decoction by the medicine room of Beijing Youan Hospital, 150 mL each time, twice daily; (2) for both qi and yin defifi ciency syndrome: Radix adenophorae 15 g, Ophiopogon japonicus 15 g, Astragalus membranaceus 15 g, Rhizoma Dioscoreae 15 g, and Massa Fermentata 10 g were decocted, 150 mL each time, twice daily.**

**^&^Detailed statistics data are presented in Appendix 2 and 3**
